# Thermal, Morphological, Mechanical, and Biodegradation Properties of Poly(L-lactide)-*b*-poly(ethylene glycol)-*b*-poly(L-lactide)/High-Density Polyethylene Blends

**DOI:** 10.3390/polym16142078

**Published:** 2024-07-21

**Authors:** Yodthong Baimark, Prasong Srihanam, Yaowalak Srisuwan

**Affiliations:** Biodegradable Polymers Research Unit, Department of Chemistry and Centre of Excellence for Innovation in Chemistry, Faculty of Science, Mahasarakham University, Mahasarakham 44150, Thailand; prasong.s@msu.ac.th (P.S.); yaowalak.s@msu.ac.th (Y.S.)

**Keywords:** poly(lactic acid), poly(ethylene glycol), block copolymer, high-density polyethylene, polymer blends

## Abstract

Polymer blends of poly(L-lactide)-*b*-poly(ethylene glycol)-*b*-poly(L-lactide) (PLLA-PEG-PLLA) and high-density polyethylene (HDPE) with different blend ratios were prepared by a melt blending method. The thermal, morphological, mechanical, opacity, and biodegradation properties of the PLLA-PEG-PLLA/HDPE blends were investigated and compared to the PLLA/HDPE blends. The blending of HDPE improved the crystallization ability and thermal stability of the PLLA-PEG-PLLA; however, these properties were not improved for the PLLA. The morphology of the blended films showed that the PLLA-PEG-PLLA/HDPE blends had smaller dispersed phases compared to the PLLA/HDPE blends. The PLLA-PEG-PLLA/HDPE blends exhibited higher flexibility, lower opacity, and faster biodegradation and bioerosion in soil than the PLLA/HDPE blends. Therefore, these PLLA-PEG-PLLA/HDPE blends have a good potential for use as flexible and partially biodegradable materials.

## 1. Introduction

Usually, conventional plastics are fossil-based and non-biodegradable, which are non-renewable and durable plastics. Although conventional plastics have a wide range of high-performance applications, they have a high carbon footprint during processing and disposal [1]. Moreover, waste from conventional plastics is also a serious pollution problem. Reduction and replacement of “fossil-based plastics” with “bio-based plastics” can decrease the carbon footprint and greenhouse gas emissions [1,2,3,4,5]. Thus, tailoring polymer blends of fossil-based and bio-based plastics is one method for reducing the carbon footprint of plastic products.

Biodegradable bioplastics are produced from bio-renewable resources and can biodegrade. Applications of biodegradable bioplastics instead of conventional plastics have steadily increased because they have a low carbon footprint during processing and disposal [1,4,5], and pollution from plastic waste can also be neglected. However, biodegradable bioplastics need an extension of their service life for some long-term applications and require improvements in some properties, such as thermal and mechanical properties.

Among synthetic biodegradable bioplastics, poly(L-lactic acid) or poly(L-lactide) (PLLA) have been considered a substitute for conventional plastics most frequently because of their biocompatibility, low cost, good processability, high mechanical strength, and availability in the market [6,7,8,9,10]. The byproducts of PLLA degradation have low toxicity; eventually, microorganisms convert them to water and carbon dioxide [11]. Therefore, PLLA has been widely used in biomedical [12] and packaging applications [13]. However, the low flexibility and high production cost of the PLLA compared to commodity plastics have restricted its wider applications [1,14,15,16,17].

Generally, the flexibility of PLLA can be improved by introducing a plasticizing effect via blending and copolymerization methods [14]. However, the PLLA-based copolymers exhibit a homogeneous single phase and have no migration effect, which reduces the effectiveness of plasticizing [18,19,20,21]. For high-molecular-weight PLLA-*b*-poly(ethylene glycol)-*b*-PLLA (PLLA-PEG-PLLA) triblock copolymers, PEG middle-blocks can act as plasticizing sites to increase the flexibility of the PLLA end-blocks [22,23]. PEG is a biocompatible and bioerodible material that is FDA-approved and can be excreted from the human body through the kidney [24]. The PLLA-PEG-PLLA exhibited higher flexibility and a faster biodegradation rate than the PLLA due to the flexible and hydrophilic PEG blocks [25]. However, research on the extent of the service life of flexible PLLA-PEG-PLLA for multiuse long-term applications is challenging.

Melt blending of PLLA with conventional plastics such as high-density polyethylene (HDPE) [26,27,28], polypropylene (PP) [29,30], and polystyrene (PS) [31], etc. has been widely investigated for use in long-term (durable) applications such as general packaging, automotive, textile, housing, and for reducing the production cost of PLLA [32]. HDPE has been extensively used for plastic products due to its low cost, high flexibility, rapid crystallization, durability, and easy processing [33]. PLLA/HDPE blends have been prepared, but they exhibit phase separation because of the low interfacial adhesion between them [28]. Some compatibilizers, such as a polyethylene-*ran*-PLLA [26], a maleic anhydride-grafted polyethylene [27], an ethylene-butyl acrylate-glycidyl methacrylate (EBA-GMA, Elvaloy PTW) [33], and a random copolymer of ethylene and glycidyl methacrylate (GMA, Lotader AX8840) [28] have been used to enhance phase compatibility and mechanical properties of the blends. However, polymer blends of PLLA-PEG-PLLA and HDPE have not yet been reported. 

In this study, we prepared PLLA-PEG-PLLA/HDPE blends without a compatibilizer by a melt blending method. The effect of the PLLA-PEG-PLLA/HDPE blend ratio on thermal properties, crystalline structures, phase compatibility, opacity, mechanical properties, wettability, and biodegradation properties of polymer blends was systematically investigated. PLLA/HDPE blends were also prepared under the same conditions for comparison.

## 2. Materials and Methods

### 2.1. Materials

The PLLA (3251D grade) with a melt flow index (MFI, measured at 190 °C under a 2.16 kg load) of 30 g/10 min was supplied by NatureWorks LLC (Waltham, MA, USA). PLLA-PEG-PLLA was synthesized by ring-opening polymerization in the bulk of the L-lactide monomer in the presence of a chain extender, as described in our previous work [34]. PEG with a molecular weight of 20,000 Da (Sigma-Aldrich, Burlington, MA, USA) and stannous octoate (95%, Sigma-Aldrich, Burlington, MA, USA) was used as the initiating system. Joncryl ADR4368 (BASF, Bangkok, Thailand) was used as a chain extender. The MFI value of the resulting PLLA-PEG-PLLA was 35 g/10 min. HDPE (HD1010J grade) with an MFI value of 26 g/10 min was purchased from PTT Chemicals (Bangkok, Thailand).

### 2.2. Preparation of PLLA/HDPE and PLLA-PEG-PLLA/HDPE Blends

PLLA/HDPE and PLLA-PEG-PLLA/HDPE blends were prepared by melt blending with a HAAKE internal mixer (model Polylab OS System, Waltham, MA, USA) at 190 °C with a 100 rpm rotor speed for 10 min. PLLA, PLLA-PEG-PLLA, and HDPE were dried in a vacuum oven at 50 °C overnight before blending. PLLA/HDPE and PLLA-PEG-PLLA/HDPE blends with blend ratios of 100/0, 80/20, 60/40, 40/60, 20/80, and 0/100 (*w*/*w*) were investigated. After being dried in a vacuum oven at 50 °C overnight, these blends were hot-pressed at 190 °C under 10 MPa compression force for 2 min using a Carver compression molding machine (model Auto CH, Wabash, IN, USA) before cooling with water-cooled plates under 10 MPa compression force for 2 min. Blended films with 0.2–0.3 mm thickness resulted.

### 2.3. Characterization of PLLA/HDPE and PLLA-PEG-PLLA/HDPE Blends

The thermal transition properties of samples were investigated using a PerkinElmer differential scanning calorimeter (DSC, model Pyris Diamond, Waltham, MA, USA) under a nitrogen flow. The previous thermal history of the sample (~5 mg) was eliminated by heating at 200 °C for 3 min before quickly quenching to 0 °C. Then, it was scanned from 0 °C to 200 °C with a heating rate of 10 °C/min and the DSC heating curves were recorded. The crystallinities of the PLLA (*PLLA−X_c_*) and HDPE (*HDPE−X_c_*) fractions were calculated using Equations (1) and (2), respectively.

*PLLA−X_c_* (%) = [(Δ*H_m_*_,*PLLA*_ − Δ*H_cc_*)/(93.6 × *W_PLLA_*)] × 100 (1)

*HDPE−X_c_* (%) = [Δ*H_m_*_,*HDPE*_/(288 × *W_HDPE_*)] × 100 (2)

where Δ*H_m_*_,*PLLA*_ and Δ*H_m_*_,*HDPE*_ are the enthalpies of melting for PLLA and HDPE, respectively. Δ*H_cc_* is the enthalpy of cold crystallization for PLLA. 93.6 J/g and 288 J/g are 100% *X_c_* of PLLA [34] and of HDPE [35], respectively. *W_PLLA_* and *W_HDPE_* are the weight fractions of PLLA and HDPE, respectively (the weight fraction of PLLA for pure PLLA-PEG-PLLA obtained from the ^1^H-NMR analysis is 0.83 [34]).

The thermal decomposition properties of samples were determined using a TA Instruments thermogravimetric analyzer (TGA, model SDT Q600, New Castle, DE, USA) under a nitrogen flow. Approximately 10 mg of the sample was scanned from 50 °C to 800 °C with a heating rate of 20 °C/min.

The crystalline structures of film samples were investigated using a Bruker wide-angle X-ray diffractometer (XRD, model D8 Advance, Karlsruhe, Germany) with CuKα radiation operating at 40 kV and 40 mA in the diffraction angle (2 theta) range of 5–30°. Scanning speed was maintained at 3 °/min.

The phase morphology of film samples was examined from the film’s cryo-fractured surfaces after treatment in liquid nitrogen using a JEOL scanning electron microscope (SEM, model JSM-6460LV, Tokyo, Japan) with an acceleration voltage of 15 kV. The sample surfaces were sputter-coated with gold before SEM observations.

The opacity of film samples was measured from the absorbance of film at a wavelength of 600 nm (*A*_600_) using a Thermo Scientific visible spectrophotometer (model Genesys 20, Loughborough, UK) [36]. Equation (3) was used to calculate the opacity of film samples.

Opacity (mm^−1^) = *A*_600_/*X* (3)

where *X* is the film’s thickness (mm).

The tensile properties of film samples with a 10 mm width were investigated using a Liyi universal testing machine (model LY-1066B, Dongguan, China) at 25 °C. A strain rate of 50 mm/min, an initial gauge length of 50 mm, and a load cell of 100 kg were used. The tensile results for each sample were averaged from ten independent tests.

The wettability of film samples was determined from the water contact angle of the film’s surfaces using a DataPhysics Instruments contact angle analyzer (model OCA 11, Filderstadt, Germany). A deionized water droplet (2.5 µL) on the film surface was captured at 15 s after dropping water. The incident angles of a water droplet were determined with OCA 11 software. The results of the water contact angle for each sample were averaged from ten independent measurements.

The biodegradation test of film samples (20 × 20 mm) involved burying them in soil for 12 months, as detailed in the literature [25]. Before burying, the film samples were dried in a vacuum oven at room temperature for 48 h before being weighed. The film sample was then packed into a nylon mesh bag with a 1.0 mm mesh size before being buried in soil at about 5.0 cm from the soil surface. The soil was watered every other day. The pH and moisture content of the soil were controlled in the pH range of 6.0–7.0 and 50–60%, respectively. The representative film samples were carefully collected every two months and washed with distilled water before drying in a vacuum oven at 50 °C for 48 h and being weighed. The weight loss in the soil of the film samples was calculated using Equation (4). The results of weight loss for each sample were averaged from three independent tests.

Weight loss in soil (%) = [(*W_i_* − *W_f_*)/*W_i_*] × 100 (4)

where *W_i_* and *W_f_* are the weights of the film samples before and after being buried in soil, respectively.

The data for film opacity, tensile strength, wettability, and biodegradation analyses were expressed as mean ± SD. Statistical analysis was performed using a one-way analysis of variance (ANOVA).

## 3. Results and Discussion

### 3.1. Thermal Transition Properties

**Figure 1:**
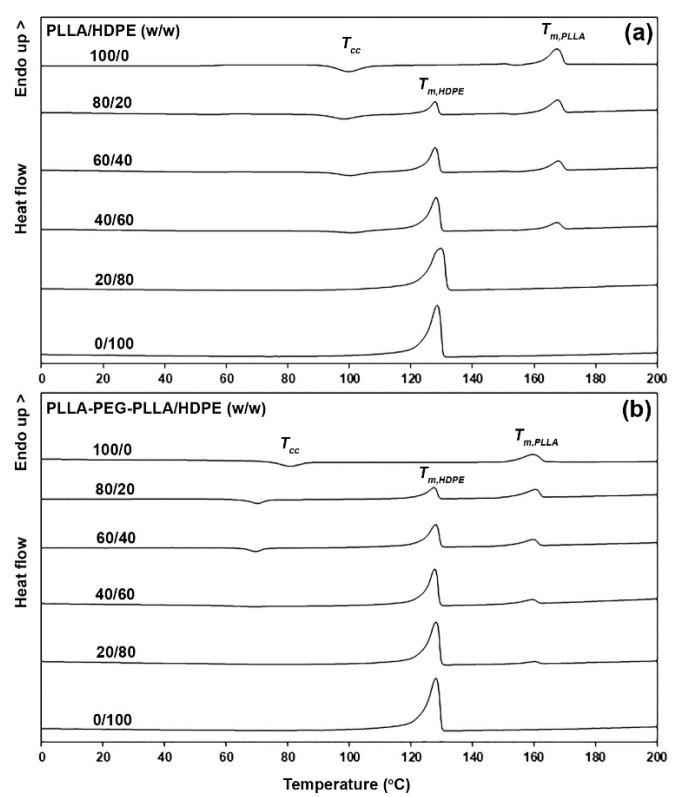
DSC heating curves of (**a**) PLLA/HDPE and (**b**) PLLA-PEG-PLLA/HDPE blends with various blend ratios (peak assignments of *T_cc_*, *T_m_*_,*PLLA*_, and *T_m_*_,*HDPE*_ as shown).

The thermal transition properties of samples were determined from DSC heating scans, as shown in Figure 1. The DSC peaks such as cold crystallization (*T_cc_*), HDPE melting (*T_m_*_,*HDPE*_), and PLLA melting (*T_m_*_,*PLLA*_) peaks were assigned, and the DSC results are summarized in Table 1. The pure PLLA and pure PLLA-PEG-PLLA had a single *T_cc_* peak at 100 °C and 81 °C, respectively. The lower-temperature *T_cc_* peak of PLLA-PEG-PLLA is attributed to its greater chain mobility for cold crystallization during the DSC heating scan. This is because the flexible PEG middle-blocks acted as plasticizing sites to enhance the chain mobility of PLLA end-blocks [22,37]. The HDPE had no *T_cc_* peak, which was attributed to it undergoing complete crystallization during DSC fast quenching from 200 °C to 0 °C before the DSC heating scan [37,38]. Therefore, all the *T_cc_* peaks in the blends could occur from PLLA’s crystallization. 

The *T_cc_* peaks of PLLA/HDPE blends were in the range of 99–101 °C, similar to the values for pure PLLA (100 °C), suggesting that the HDPE blending did not affect the crystallization of PLLA. However, the *T_cc_* peak of the PLLA-PEG-PLLA matrix dramatically shifted down from 81 °C to 69–70 °C when HDPE was incorporated, suggesting that HDPE blending enhanced the crystallization ability of PLLA-PEG-PLLA through the nucleating effect. The shifting to a lower temperature of *T_cc_* peaks suggested that the interfacial adhesion between PLLA end-blocks and HDPE could be improved with the PEG middle-blocks [26]. For the PLLA-PEG-PLLA-based blends, the dispersed HDPE particles may have acted as a nucleating agent to reduce the nucleation induction period and increased the number of nucleating sites for the crystallization of the PLLA-PEG-PLLA matrix [37]. 

The *T_m_*_,*HDPE*_ peak of pure HDPE was at 128 °C. The *T_m_*_,*PLLA*_ peaks of pure PLLA and pure PLLA-PEG-PLLA were at 167 °C and 160 °C, respectively. The pure PLLA-PEG-PLLA had a *T_m_* peak at a lower temperature than the pure PLLA, which may have been due to the PEG middle-blocks causing the production of imperfect crystallites in the PLLA end-blocks. All the PLLA/HDPE and PLLA-PEG-PLLA/HDPE blends exhibited both the *T_m_*_,*HDPE*_ and *T_m_*_,*PLLA*_ peaks. These peaks were nearly the same value as each pure component, suggesting that each component in the blends was immiscible [35].

The crystallinity of the PLLA fraction (*X_c_*_,*PLLA*_) is also reported in Table 1, showing that the *X_c_*_,*PLLA*_ values of pure PLLA and PLLA/HDPE blends were in the range of 10.0–12.0%. This supports the hypothesis that HDPE blending did not affect PLLA crystallization, corresponding to the *T_cc_* peaks of blends, which did not shift down. The *X_c_*_,*PLLA*_ of PLLA-PEG-PLLA/HDPE blends increased as the HDPE content increased. As the PLLA-PEG-PLLA was a blend matrix for 80/20 and 60/40 (*w*/*w*) PLLA-PEG-PLLA/HDPE blends, the dispersed HDPE particles could act as a nucleating agent according to the displacement of the *T_cc_* peak towards a lower temperature, as described above. It should be noted that the *X_c_*_,*PLLA*_ of PLLA-PEG-PLLA/HDPE blends still increased when the PLLA-PEG-PLLA was a dispersed phase for 40/60 and 20/80 (*w*/*w*) PLLA-PEG-PLLA/HDPE blends, suggesting that the HDPE blend matrix can also accelerate PLLA crystallization of dispersed PLLA-PEG-PLLA phases. This was supported by the *T_cc_* value of the 40/60 (*w*/*w*) PLLA-PEG-PLLA/HDPE blend being 69 °C, which was lower than that of the pure PLLA-PEG-PLLA (81 °C). The crystallinity of the HDPE fraction (*X_c_*_,*HDPE*_) calculated from Equation (2) for both the PLLA/HDPE and PLLA-PEG-PLLA/HDPE blend families increased with increasing HDPE content. This may be explained by the HDPE crystallinity increased in accordance with the number of HDPE chains. 

### 3.2. Thermal Decomposition Properties

**Figure 2:**
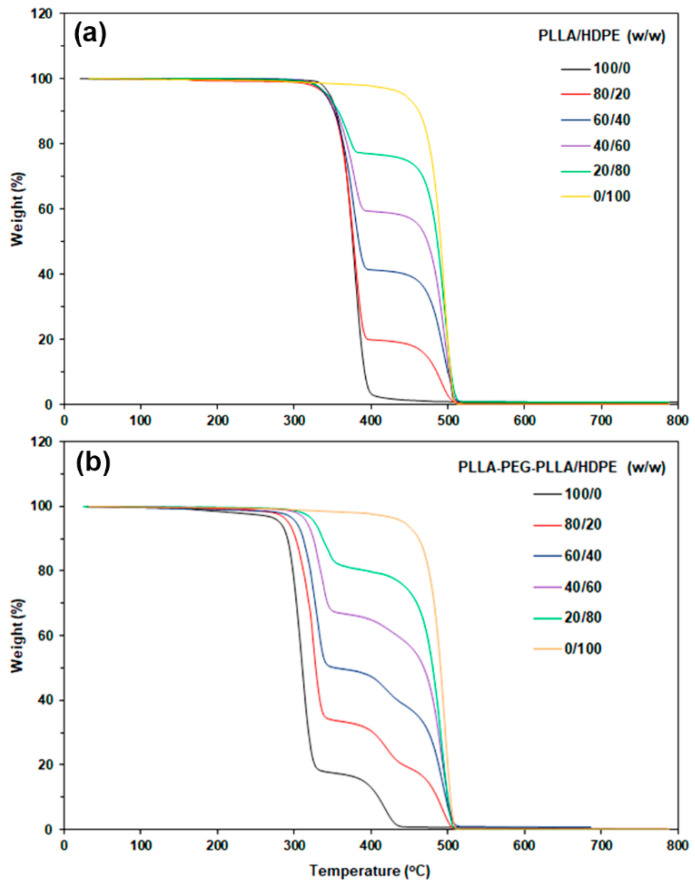
TG thermograms of (**a**) PLLA/HDPE and (**b**) PLLA-PEG-PLLA/HDPE blends with various blend ratios.

The TGA test was used to investigate the thermal decomposition properties of samples by measuring the weight loss of samples as a function of temperature in a nitrogen atmosphere. Figure 2a,b shows TG thermograms of PLLA/HDPE and PLLA-PEG-PLLA/HDPE blends, respectively. In Figure 2a, both the pure PLLA (black line) and pure HDPE (yellow line) exhibited a single thermal-decomposition stage in the range of 300–450 °C and 450–520 °C, respectively. All the PLLA/HDPE blends clearly exhibited two thermal-decomposition stages due to the PLLA and HDPE decompositions. The weight loss in the HDPE decomposition stage was directly related to the HDPE content. It was found that the temperature ranges of thermal decomposition for each PLLA and HDPE component in the PLLA/HDPE blends were similar to those of pure PLLA and pure HDPE, respectively.

**Figure 3:**
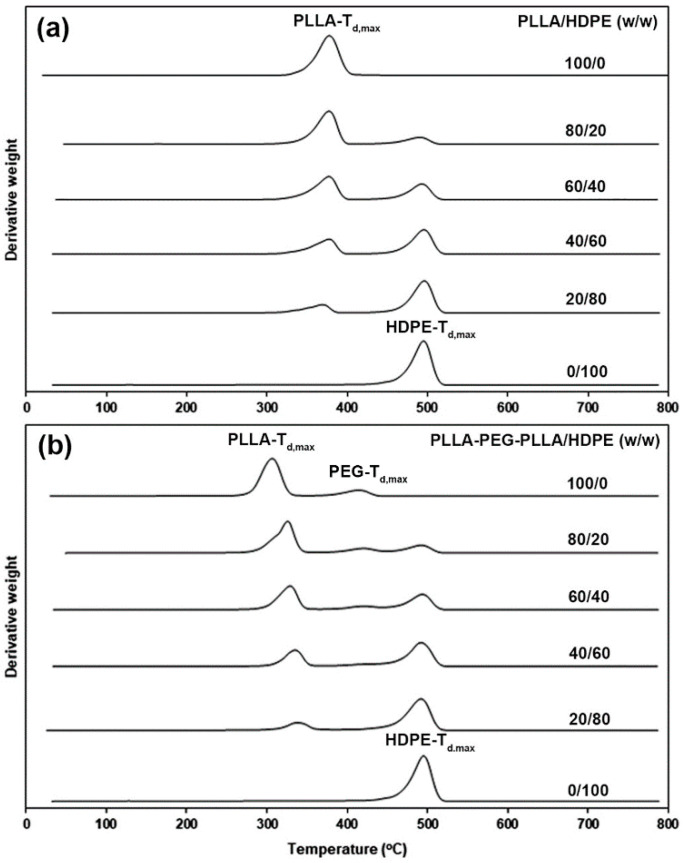
DTG thermograms of (**a**) PLLA/HDPE and (**b**) PLLA-PEG-PLLA/HDPE blends with various blend ratios (peak assignments of *PLLA-T_d_*_.*max*_, *HDPE-T_d_*_.*max*_, and *PEG-T_d_*_.*max*_ as shown).

The TG thermogram of pure PLLA-PEG-PLLA presented in Figure 2b (black line) clearly shows two thermal-decomposition stages of the PLLA end-blocks and PEG middle-blocks in the ranges of 250–350 °C and 350–450 °C, respectively [23,34]. All the PLLA-PEG-PLLA/HDPE blends showed three thermal-decomposition stages attributed to decompositions of PLLA end-blocks, PEG middle-blocks, and HDPE phases. It is very interesting that the thermal-decomposition stage of PLLA end-blocks significantly shifted up to a higher temperature with the addition of HDPE and increasing HDPE content. This result will be supported later by the results from the DTG thermogram analysis shown in Figure 3.

Figure 3a,b show DTG thermograms of PLLA/HDPE and PLLA-PEG-PLLA/HDPE blends, respectively. We can see the DTG peaks, which are decomposition temperatures at maximum rate (*T_d_*_.*max*_) of PLLA (*PLLA-T_d.max_*), PEG (*PEG-T_d.max_*), and HDPE (*HDPE-T_d.max_*). The resulting *T_d.max_* values are summarized in Table 2. The *T_d.max_* values of pure PLLA and pure HDPE were 379 °C and 495 °C, respectively. For PLLA/HDPE blends, the *PLLA-T_d.max_* and *HDPE-T_d.max_* values were similar in the ranges of 375–378 °C and 493–495 °C, respectively. This supported the conclusion that blending PLLA with HDPE did not affect the thermal decomposition of each pure component, as described in the literature [35]. 

The pure PLLA-PEG-PLLA had its *PLLA-T_d.max_* peak at 310 °C and *PEG-T_d.max_* at 417 °C. It was found that the *PLLA-T_d.max_* peak of the PLLA-PEG-PLLA/HDPE blend dramatically shifted up to 325 °C by blending with 20 %wt HDPE, which was 15 °C higher than the pure PLLA-PEG-PLLA (310 °C). Moreover, the *PLLA-T_d.max_* value of blends steadily shifted up to a higher temperature with increasing HDPE content. The results confirmed that the HDPE blending enhanced the thermal stability of PLLA end-blocks. It has been reported that the PLLA/polystyrene [39] and compatibilized PLLA/HDPE blends [40] showed higher thermal stability than the pure PLLA due to the presence of intermolecular interactions in the blends. The *PEG-T_d.max_* and *HDPE-T_d.max_* values of the PLLA-PEG-PLLA/HDPE blends were similar to those of each pure component and were in the ranges of 417–418 °C and 492–494 °C, respectively. Thus, the addition of HDPE improved the thermal stability and increased the processing window of PLLA-PEG-PLLA, whereas the thermal stability of HDPE did not change.

### 3.3. Crystalline Structures

**Figure 4:**
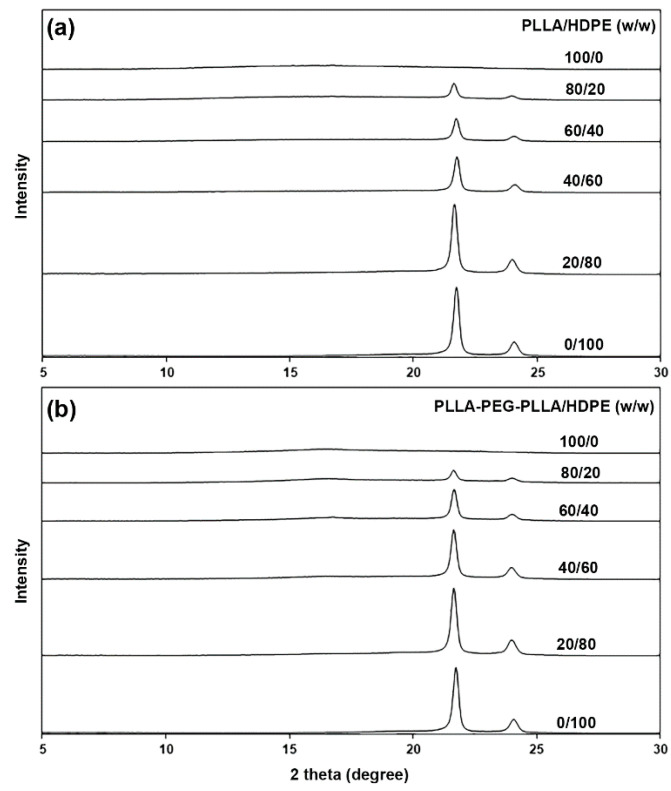
XRD patterns of (**a**) PLLA/HDPE and (**b**) PLLA-PEG-PLLA/HDPE blends with various blend ratios.

The crystalline structures of the film samples were determined from XRD patterns, as shown in Figure 4. The pure HDPE had XRD peaks at 2-theta of 21.6° and 24.1° attributed to HDPE crystallites [27,35]. All PLLA/HDPE and PLLA-PEG-PLLA/HDPE blends also exhibited these peaks at 2-theta of 21.6° and 24.1°, which indicated that the PLLA or PLLA-PEG-PLLA blending did not change the crystalline structures of HDPE. As would be expected, the intensity of these peaks steadily increased as the HDPE content increased, indicating that the crystallinity of HDPE increased.

**Figure 5:**
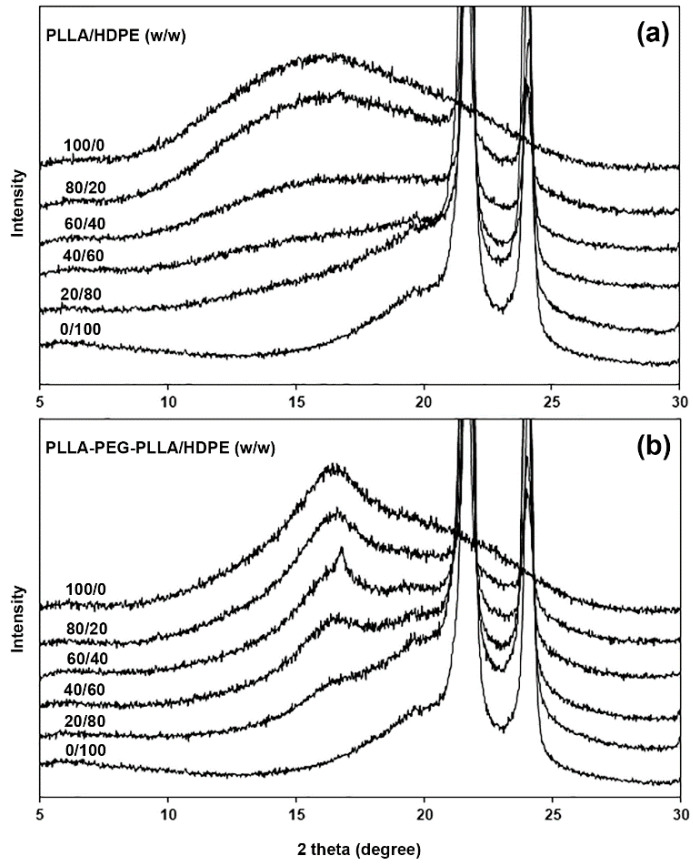
Expanded XRD patterns of (**a**) PLLA/HDPE and (**b**) PLLA-PEG-PLLA/HDPE blends with various blend ratios.

The expanded XRD patterns in Figure 5 were used to study the crystalline structures of pure PLLA, pure PLLA-PEG-PLLA, and its blends. The pure PLLA and PLLA/HDPE blends had no XRD peak of PLLA’s crystallites, suggesting that all the PLLA phases were completely amorphous. The pure PLLA-PEG-PLLA had a broad XRD peak at 2-theta of 16.9° due to the crystallites of PLLA end-blocks [34,38]. All the PLLA-PEG-PLLA/HDPE blends also showed a broad XRD peak at 2-theta of 16.9°, which indicated that the HDPE blending did not affect the crystalline structures of PLLA end-blocks. The peak intensity at 2-theta of 16.9° of PLLA-PEG-PLLA/HDPE blends decreased with decreasing PLLA-PEG-PLLA content.

### 3.4. Phase Morphology

**Figure 6:**
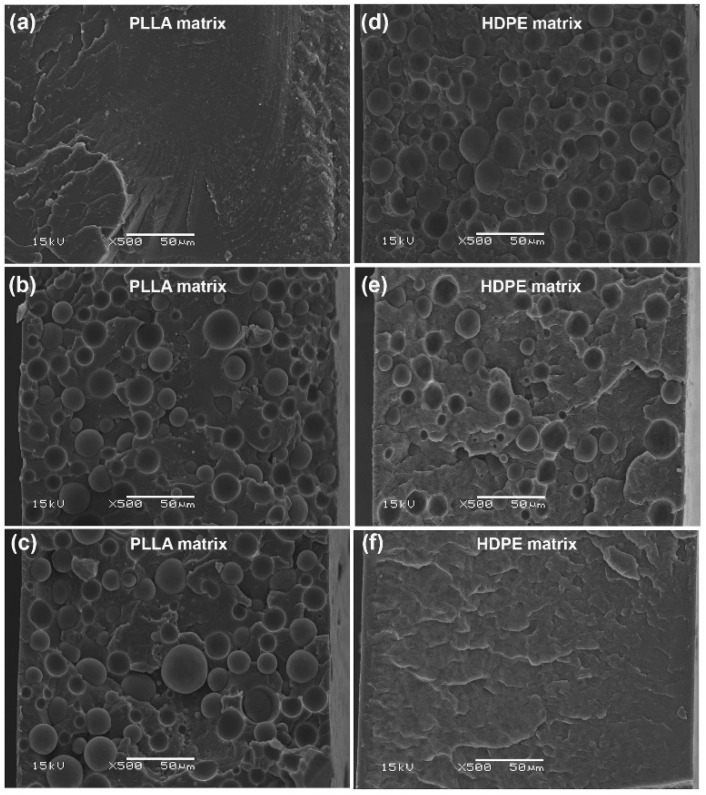
SEM images of cryo-fractured surfaces of (**a**) pure PLLA film and PLLA/HDPE blended films with HDPE contents of (**b**) 20 %wt, (**c**) 40 %wt, (**d**) 60 %wt, and (**e**) 80 %wt as well as (**f**) pure HDPE film (all bar scales = 50 µm).

**Figure 7:**
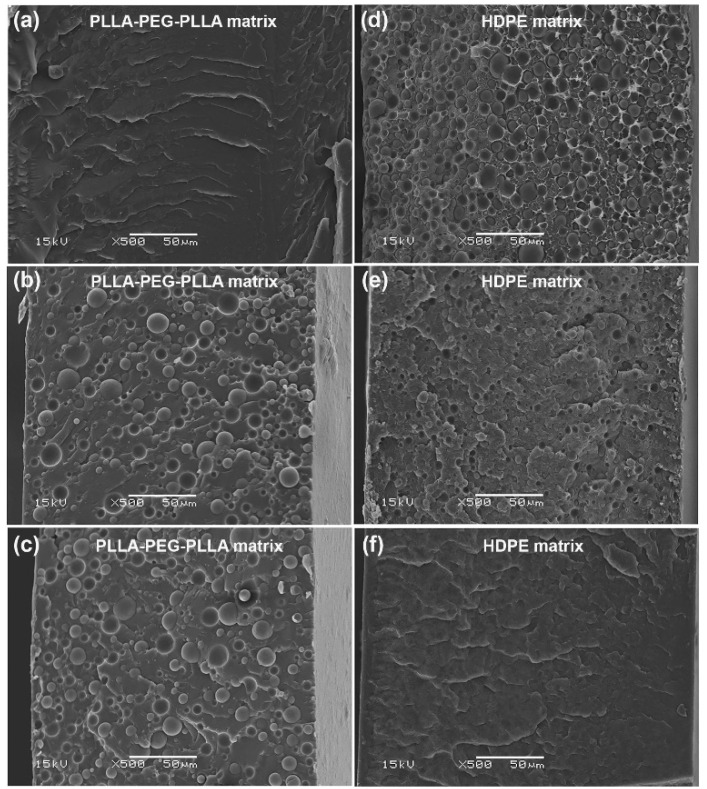
SEM images of cryo-fractured surfaces of (**a**) pure PLLA-PEG-PLLA film and PLLA-PEG-PLLA/HDPE blended films with HDPE contents of (**b**) 20 %wt, (**c**) 40 %wt, (**d**) 60 %wt, and (**e**) 80 %wt as well as (**f**) pure HDPE film (all bar scales = 50 µm).

The phase morphology of the blended films was investigated from SEM images of cryo-fractured surfaces of PLLA/HDPE and PLLA-PEG-PLLA/HDPE blended films, as illustrated in Figure 6 and Figure 7, respectively. The pure PLLA film in Figure 6a showed an almost smooth fractured surface indicative of its brittle character, whereas the pure HDPE film in Figure 6f had a rougher fractured surface indicative of its flexible character. All the PLLA/HDPE blends exhibited droplet-like morphology, indicating immiscibility between blend components [26,28,32]. The 80/20 and 60/40 (*w*/*w*) PLLA/HDPE blended films in Figure 6b,c, respectively, showed dispersed HDPE particles in the PLLA matrix. Whereas, the 40/60 and 20/80 (*w*/*w*) PLLA/HDPE blended films in Figure 6d,e, respectively, showed dispersed PLLA particles in the HDPE matrix. The cryo-fractured PLLA-PEG-PLLA film depicted in Figure 7a had a rough surface, indicating that it was flexible. The dispersed HDPE particles in the PLLA-PEG-PLLA matrix were observed for the 80/20 and 60/40 (*w*/*w*) PLLA-PEG-PLLA/HDPE blended films, as shown in Figure 7b,c, respectively. Whereas the dispersed PLLA-PEG-PLLA particles in the HDPE matrix were observed for the 40/60 and 20/80 (*w*/*w*) PLLA-PEG-PLLA/HDPE blended films, as shown in Figure 7d,e, respectively.

The most dispersed phases in all the PLLA/HDPE blends appeared to have larger particles than those in all the PLLA-PEG-PLLA/HDPE blends. The results indicate that phase compatibility between the PLLA-PEG-PLLA and HDPE was better than between the PLLA and HDPE. The reduction in dispersed particle size in the blends suggests an improvement in phase compatibility between blend components [26,28,41]. The addition of appropriate compatibilizers can reduce dispersed particle sizes by reducing surface tension between the PLLA and HDPE phases [32,33]. Therefore, the PEG middle-blocks may act as compatibilizing sites between PLLA end-blocks and HDPE to produce the partially miscible PLLA-PEG-PLLA/HDPE blends [32]. The SEM analysis may also be used to support the hypothesis that the crystallization properties and thermal stability of PLLA end-blocks from DSC and TGA analyses, respectively, improved because the PEG middle-blocks enhanced compatibility between the PLLA end-blocks and HDPE.

### 3.5. Film Opacity

**Figure 8:**
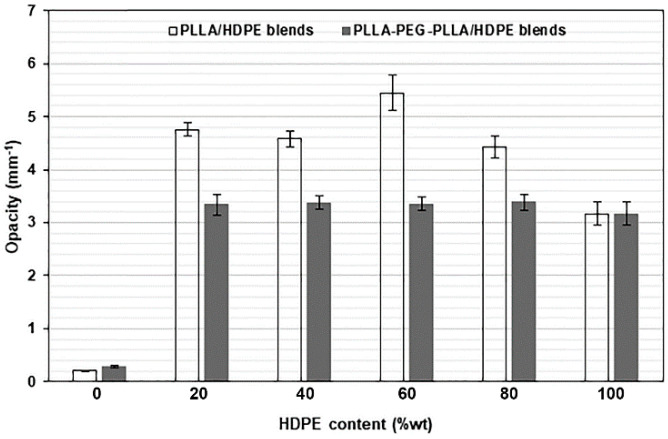
Opacity of PLLA/HDPE and PLLA-PEG-PLLA/HDPE blended films with various HDPE contents.

The opacity of film samples was calculated from Equation (3), and the obtained values are compared in Figure 8. The pure PLLA, pure PLLA-PEG-PLLA, and pure HDPE films had opacity values of 0.2061, 0.2863, and 3.1720 mm^−1^, respectively. The opacity values of these pure films depended upon their crystallinities [42], corresponding to the intensity of XRD peaks (see Figure 4 and Figure 5). The pure PLLA film had no XRD peaks attributable to it and was judged to be completely amorphous. Thus, the pure PLLA film had the lowest opacity (or the highest transparency), whereas the pure PLLA-PEG-PLLA film had a broad XRD peak at 2-theta of 16.9°; therefore, the pure PLLA-PEG-PLLA film was more opaque than the pure PLLA films. The XRD peak intensities of pure HDPE film were the highest. Thus, the pure HDPE film had the highest opacity.

**Figure 9:**
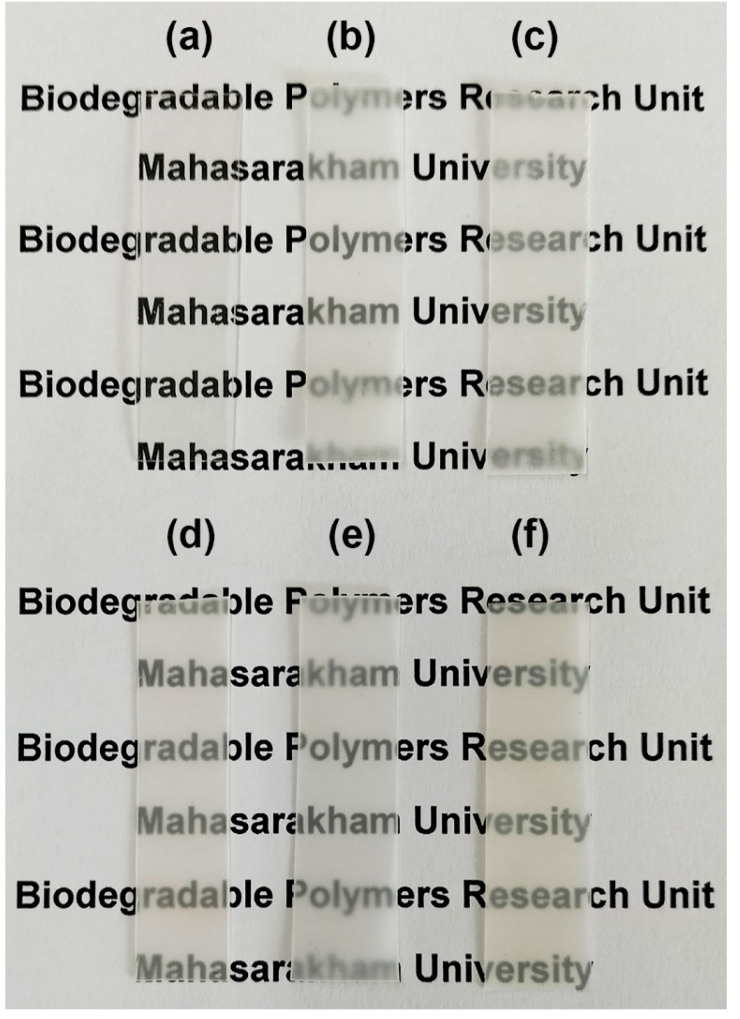
Photographs of (**a**) pure PLLA film and PLLA/HDPE blended film with HDPE contents of (**b**) 20 %wt, (**c**) 40 %wt, (**d**) 60 %wt, and (**e**) 80 %wt as well as (**f**) pure HDPE film.

**Figure 10:**
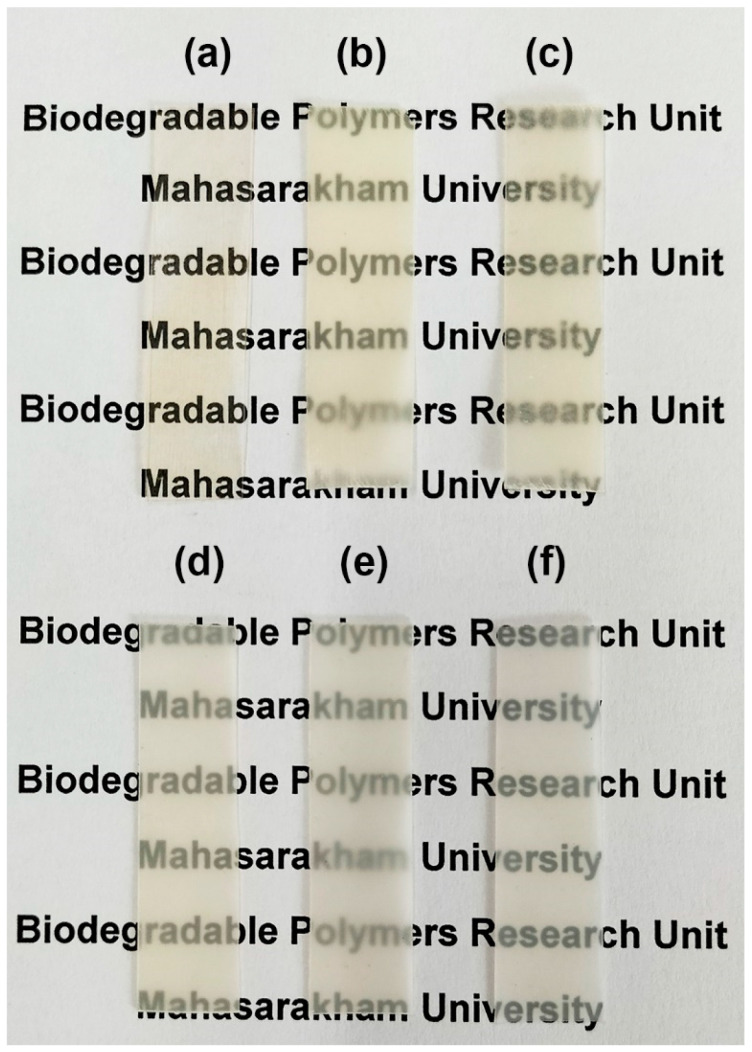
Photographs of (**a**) pure PLLA-PEG-PLLA film and PLLA-PEG-PLLA/HDPE blended film with HDPE contents of (**b**) 20 %wt, (**c**) 40 %wt, (**d**) 60 %wt, (**e**) 80 %wt as well as (f) pure HDPE film.

In Figure 8, it is shown that the opacity of all films of PLLA/HDPE and PLLA-PEG-PLLA/HDPE blends was higher than each of the pure films because these blends were immiscible and consisted of continuous and dispersed phases, as described above in the SEM analysis. Generally, two-phase polymer blends containing micro-scale dispersed phases will be opaque. It should be noted that all the PLLA-PEG-PLLA/HDPE blended films showed lower film opacity than all the PLLA/HDPE blended films. This may be due to the dispersed particles in the PLLA-PEG-PLLA/HDPE blended films having smaller sizes, corresponding to the above SEM analysis. However, all the blended films were still highly transparent, as shown in Figure 9 and Figure 10 for the PLLA/HDPE and PLLA-PEG-PLLA/HDPE blended films, respectively. The words written under these blended films were still easily visible and readable. Therefore, these blends could be useful as packaging materials since the product characteristics can still be observed.

### 3.6. Tensile Properties

**Figure 11:**
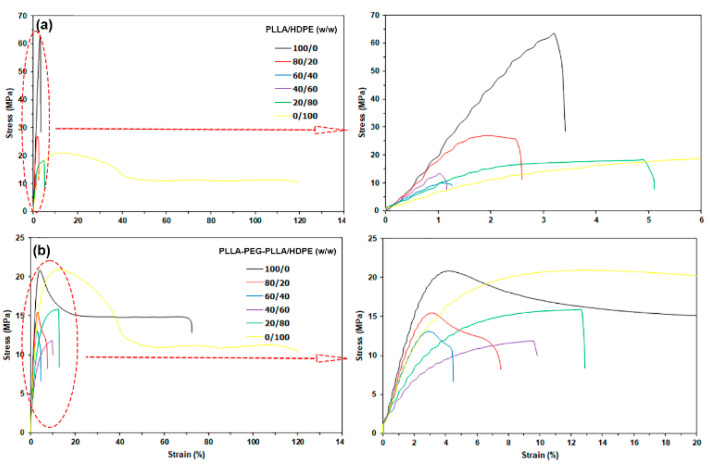
Tensile curves of (**a**) PLLA/HDPE and (**b**) PLLA-PEG-PLLA/HDPE blended films with various blend ratios (their expanded initial tensile curves were indicated by red arrows).

**Figure 12:**
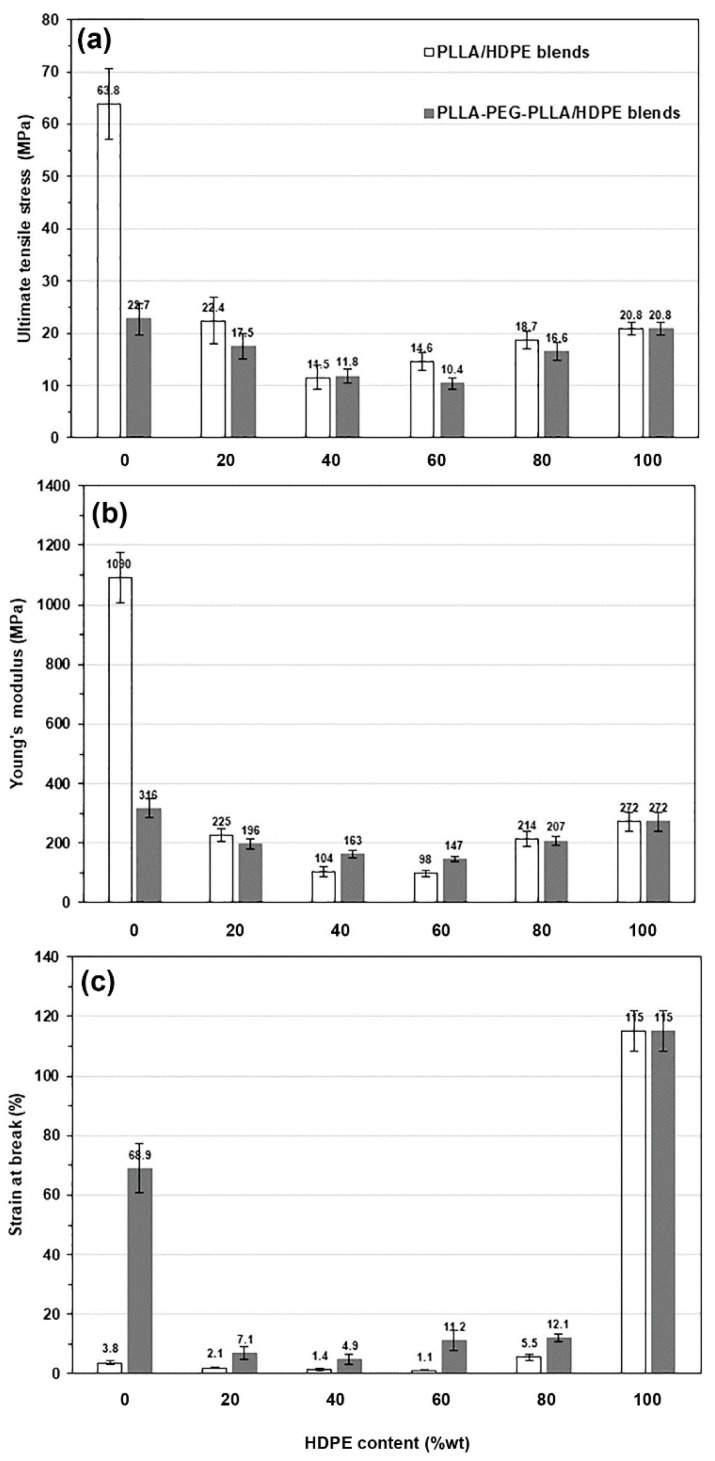
Tensile properties of PLLA/HDPE and PLLA-PEG-PLLA/HDPE blended films with various HDPE contents: (**a**) ultimate tensile stress, (**b**) Young’s modulus, and (**c**) strain at break (average values as shown).

The tensile properties, such as ultimate tensile stress, Young’s modulus, and strain at break of the film samples were measured to evaluate their mechanical properties. Figure 11 shows selected tensile curves and their expanded curves for the film samples. The average tensile properties are presented and compared in Figure 12. The pure PLLA film [Figure 11a, black line] exhibited brittle character with an ultimate tensile stress of 63.8 MPa, a Young’s modulus of 1090 MPa, and a strain at break of 3.8%, whereas the pure HDPE film [Figure 11a, yellow line] had a yield point assigned to flexible character with an ultimate tensile stress of 20.8 MPa, a Young’s modulus of 272 MPa, and a strain at break of 115%. In Figure 12, the ultimate tensile stress, Young’s modulus, and strain at break of the PLLA-based film matrix are seen to have decreased as the 20%wt and 40%wt HDPE were incorporated. However, these tensile properties of HDPE-based films also decreased when the 20%wt and 40%wt PLLA were blended, and according to the literature [35], this was because they were immiscible.

The pure PLLA-PEG-PLLA film [Figure 11b black line] had a yield point attributed to its flexible character with an ultimate tensile stress of 22.7 MPa, a Young’s modulus of 316 MPa, and a strain at break of 68.9%. The pure PLLA-PEG-PLLA exhibited higher flexibility than the pure PLLA because the flexible PEG middle-blocks induced a plasticizing effect [22,23]. In Figure 12, it is shown that the PLLA-PEG-PLLA/HDPE blended films exhibited lower tensile properties than each pure component. The tensile properties of immiscible blends usually exhibit lower values than each origin component [32]. 

The addition of compatibilizers to PLLA/HDPE blends can improve the tensile properties of the blends by enhancing the interfacial adhesion between the PLLA and HDPE [26,28]. From SEM analysis in this work, although the PLLA-PEG-PLLA/HDPE blends exhibited better phase compatibility than the PLLA/HDPE blends, the interfacial adhesion between the PLLA-PEG-PLLA and HDPE may not have been enough to improve the tensile properties of the blends. However, all the PLLA-PEG-PLLA/HDPE blended films still showed higher strain at break than the PLLA/HDPE blended films for the same HDPE content, indicating that the PLLA-PEG-PLLA/HDPE blended films were still more flexible than the PLLA/HDPE blended films.

### 3.7. Wettability

**Figure 13:**
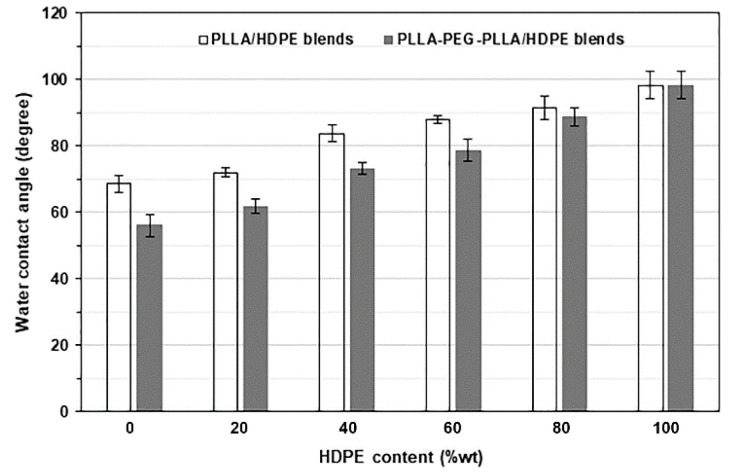
Water contact angles of PLLA/HDPE and PLLA-PEG-PLLA/HDPE blended films with various HDPE contents.

The wettability of film samples was determined by the water contact angle on the surface of the films. The effect of HDPE content on the water contact angle of blended films is shown in Figure 13. The water contact angles of pure PLLA, pure PLLA-PEG-PLLA, and pure HDPE films were 68.6°, 56.0°, and 98.2°, respectively. The results showed that the wettability of film samples was in this ranked order: PLLA-PEG-PLLA > PLLA > HDPE. The hydrophilic PEG middle-blocks enhanced the wettability of PLLA-PEG-PLLA [43]. It has been reported that the pure HDPE had higher hydrophobicity than the pure PLLA [33,35]. As would be expected, the water contact angle of blended films steadily increased (wettability decreased) with increasing HDPE content because of the high hydrophobicity of HDPE. The PLLA-PEG-PLLA/HDPE blended films had a lower water contact angle (higher hydrophilicity) than the PLLA/HDPE blended films for the same HDPE content. The results support the conclusion that the wettability of PLLA-PEG-PLLA/HDPE blends was higher than the PLLA/HDPE blends, and the wettability of blended film depended upon the HDPE content.

### 3.8. Biodegradation Test

**Figure 14:**
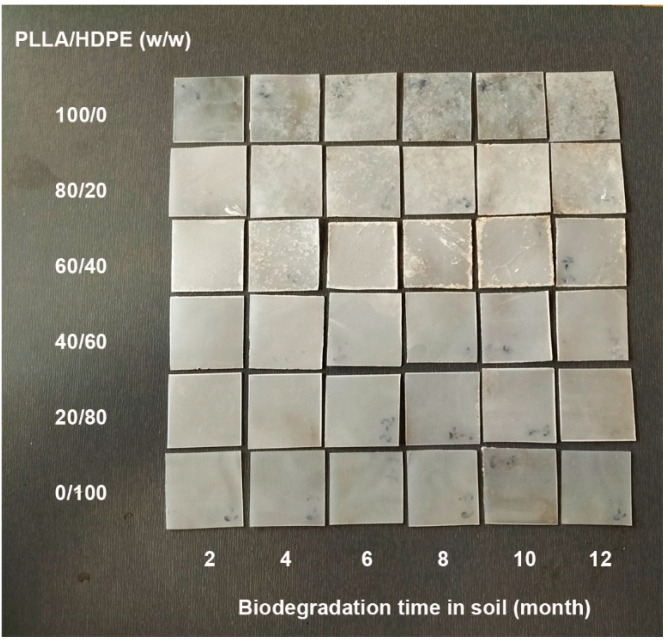
Photograph of PLLA/HDPE blended films with various blend ratios after being buried in soil for 12 months.

**Figure 15:**
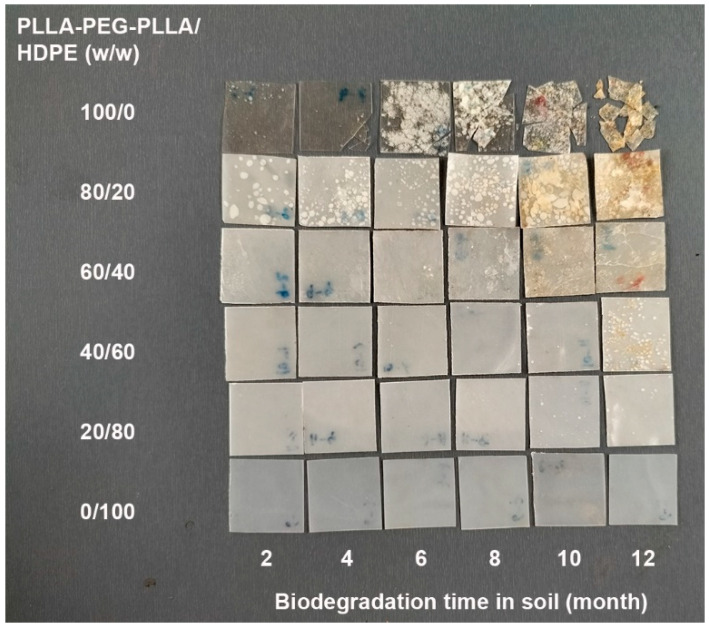
Photograph of PLLA-PEG-PLLA/HDPE blended films with various blend ratios after being buried in soil for 12 months.

The biodegradation test of film samples was performed in soil for 12 months. Figure 14 and Figure 15, respectively, show photographs of PLLA/HDPE and PLLA-PEG-PLLA/HDPE blended films after being buried in soil for different lengths of time. At the burial time of 12 months, the pure PLLA and pure HDPE films (Figure 14) did not significantly change, whereas the pure PLLA-PEG-PLLA film was broken into small pieces and clearly disintegrable in compost (Figure 15). Thus, the pure PLLA-PEG-PLLA had faster biodegradation and bioerosion rates than both the PLLA and HDPE. This was due to the pure PLLA-PEG-PLLA with higher hydrophilicity having a faster biodegradation rate [25,43,44] and the PEG blocks being bioerodible [24]. It is well known that PLLA has a slow biodegradation rate [25], whereas HDPE is non-biodegradable [1,32,35]. All the buried PLLA/HDPE blended films did not change their characteristics, whereas the PLLA-PEG-PLLA/HDPE blended films showed some biodegradation and bioerosion characteristics, such as many voids in film matrices and film breaking, especially blended films with high PLLA-PEG-PLLA content.

**Figure 16:**
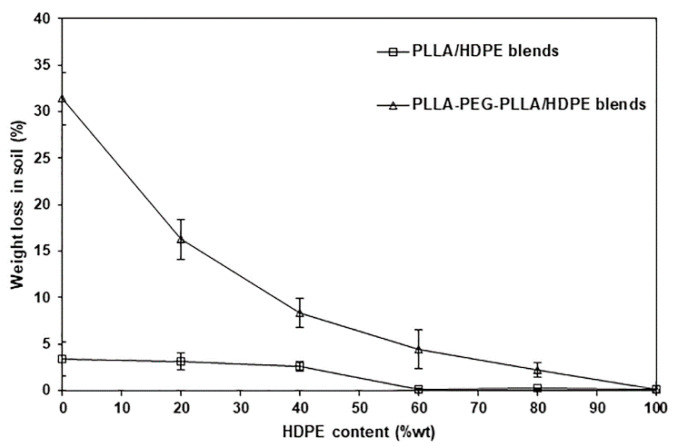
Weight losses of PLLA/HDPE and PLLA-PEG-PLLA/HDPE blended films with various HDPE contents after being buried in soil for 12 months.

The biodegradation behaviors of film samples were also determined, and the weight loss was compared after being buried in soil for 12 months. Figure 16 shows weight loss at 12-month buried time as a function of HDPE content. After being buried in soil for 12 months, the pure PLLA and pure PLLA-PEG-PLLA films had weight losses of 3.43% and 31.40%, respectively, whereas the weight loss of the pure HDPE film was less than 0.1%. This supported the view that the pure PLLA-PEG-PLLA film degraded more rapidly than the pure PLLA, whereas the pure HDPE film did not degrade. This may be due to the fact that the PLLA and PEG blocks are biodegradable [7] and bioerodible [24], respectively. Cleavage or scission of the PLLA blocks can occur as a result of biodegradation. The PEG blocks were mechanically eroded by biological processes known as bioerosion, which makes PEG soluble and permits absorption into the surrounding environment [45]. The weight loss of both the blended film families at 12-month burial time decreased as the HDPE content decreased because HDPE is non-biodegradable. Therefore, the weight loss in the soil of blended films decreased as the HDPE content increased. It could be proposed that the service life of the PLLA-PEG-PLLA/HDPE blends can be adjusted by varying the blend ratio.

## 4. Conclusions

In this work, the effect of blend ratio on the thermal properties, crystalline structures, phase compatibility, opacity, tensile properties, wettability, and biodegradation of PLLA-PEG-PLLA/HDPE blends was investigated compared to PLLA/HDPE blends. It was found that the PEG middle-blocks enhanced the crystallization properties and thermal stability of the PLLA end-blocks in PLLA-PEG-PLLA/HDPE blends, but these properties of the PLLA phases in PLLA/HDPE blends did not change. The crystalline structures of PLLA end-blocks and HDPE did not change by blending. The dispersed particles in PLLA-PEG-PLLA/HDPE blend matrices had smaller sizes than in PLLA/HDPE blend matrices, suggesting the PEG middle-blocks enhanced phase compatibility between the PLLA-PEG-PLLA and HDPE. The film’s opacity of all the blends was higher than for each pure component. The PLLA-PEG-PLLA/HDPE blended films exhibited lower opacity (higher transparency) than the PLLA/HDPE blended films. The tensile properties of film samples exhibited lower values than did each pure component. However, the PLLA-PEG-PLLA/HDPE blends still had higher flexibility than the PLLA/HDPE blends. The wettability of film samples and the reduction of weight after being buried in soil for 12 months decreased from increasing the HDPE content for both the PLLA-PEG-PLLA/HDPE and PLLA/HDPE blend families. The reduction of the films’ weight in soil for 12 months was ranked from highest to lowest in the order of pure PLLA-PEG-PLLA, PLLA-PEG-PLLA/HDPE blends, pure PLLA, PLLA/HDPE blends, and pure HDPE. It can be concluded that the crystallization ability and thermal stability of PLLA-PEG-PLLA can be improved, as well as that its weight loss in the soil can be decreased by HDPE blending. The PLLA-PEG-PLLA/HDPE blends may be used as flexible and partially biodegradable materials in long-term applications.

## Figures and Tables

**Table 1:** Thermal transition properties of PLLA/HDPE and PLLA-PEG-PLLA/HDPE blends.

Blend Ratio (*w*/*w*)	*T_cc_* (°C)	*T_m_*_,*HDPE*_ (°C)	*T_m_*_,*PLLA*_ (°C*)*	*X_c_*_,*HDPE*_ (%)	*X_c_*_,*PLLA*_ (%)
PLLA/HDPE					
100/0	100	-	167	-	10.5
80/20	99	128	167	47.0	12.0
60/40	100	128	167	50.2	10.0
40/60	101	128	167	50.4	11.8
20/80	-	129	-	55.0	-
0/100	-	128	-	60.5	-
PLLA-PEG-PLLA/HDPE					
100/0	81	-	160	-	11.6
80/20	70	128	160	51.0	20.4
60/40	70	128	160	52.2	21.7
40/60	69	128	160	56.4	32.5
20/80	-	128	160	56.6	38.6
0/100	-	128	-	60.5	-

**Table 2:** *T_d_*_.*max*_ values of PLLA/HDPE and PLLA-PEG-PLLA/HDPE blends.

Blend Ratio (*w*/*w*)	*HDPE-T_d_*_.*max*_ (°C)	*PEG-T_d_*_.*max*_ (°C)	*HDPE-T_d_*_.*max*_ (°C)
PLLA/HDPE			
100/0	379	-	-
80/20	376	-	493
60/40	378	-	493
40/60	377	-	495
20/80	375	-	494
0/100	-	-	495
PLLA-PEG-PLLA/HDPE			
100/0	310	417	-
80/20	325	417	493
60/40	328	418	494
40/60	334	-	492
20/80	339	-	493
0/100	-	-	495

## Data Availability

Data are contained within the article.
